# Supplementary material for: Reconstructing Sordida subcomplex (Hemiptera, Reduviidae, Triatominae) phylogeny across species distribution range
Source: Mem Inst Oswaldo Cruz. 2025 Dec 15;120:e250088. doi: 10.1590/0074-02760250088 (PMC12711208; doi:10.1590/0074-02760250088)
Supplement: Supplementary material [file 1678-8060-mioc-120-e250088-s.pdf]

TABLE I

Geographic origin of individuals, depicting the mitochondrial haplotype, latitude and longitude, locality and country (when the coordinates used in the map were not the exact ones, are specifically mentioned in the table). Species as are indicated in NCBI and the reference paper. In red appear the GenBank sequences erroneously identified.

Haplotype	Lineage	Latitud	Longitud	Geographic origin	NCBI species	Reference
Hap_1	*T. infestans*	Not on the map			*T. sordida*	(1)
Hap_2	*T. rubrovaria*	Not on the map			*T. rubrovaria*	(1)
Hap_3	*T. guasayana*	Not on the map		Argentina, Córdoba, Sobremonte.		This paper
Hap_4	*T. guasayana*	Not on the map		Argentina, Córdoba, Sobremonte.		This paper
Hap_5	*T.sordida.s.s*	-22,86108	-57,175868	Paraguay, Concepcion, Zona 3		This paper
Hap_6	*T.sordida.s.s*	-23,8833	-56,5667	Paraguay, San Pedro, Santa Rosa, Centro		This paper
Hap_7	*T.sordida.s.s*	-26	-60	Argentina, Chaco, El Colchon		This paper
Hap_8	*T.sordida.s.s*	-24,568524	-56,769182	Paraguay, San Pedro, Itacurubí del Rosario, Rios Rugua		This paper
Hap_9	*T.sordida.s.s*	-24	-57	Paraguay, San Pedro, San Pedro, Yatebu Rugua		This paper
Hap_10	*T.sordida.s.s*	-24	-57	Paraguay, San Pedro, Itacurubí del Rosario, Campo Virgen		This paper
Hap_11	*T.sordida.s.s*	-25	-57	Paraguay, San Pedro, Itacurubí del Rosario, Rios Rugua		This paper
Hap_12	*T.sordida.s.s*	-12,51	-46,33	Brazil, Tocantins, Combinado		This paper
Hap_13	*T.sordida.s.s*	-15	-56	Brazil, Mato Grosso, Varzea Grande		This paper
Hap_14	*T.sordida.s.s*	-16,74	-43,86	Brazil, Minas Gerais, Montes Claros		This paper
Hap_15	*T.sordida.s.s*	-18	-44	Brazil, Minas Gerais, Buenopolis (Not exact locality)	*T. sordida*	(2)
Hap_16	*T.sordida.s.s*	-19	-63	Bolivia, Santa Cruz, Izozog		This paper
Hap_16	*T.sordida.s.s*	-16	-41	Brazil, Minas Gerais, Itaobim		This paper
Hap_16	*T.sordida.s.s*	-17,87277778	-44,18	Brazil, Minas Gerais, Buenopolis (Not exact locality)	*T. sordida*	(2)
Hap_16	*T.sordida.s.s*	-21	-53	Brazil, Mato Grosso do Sul, Brasilandia (Not exact coordinates)	*T. sordida*	(3)
Hap_17	*T.sordida.s.s*	-18	-44	Brazil, Minas Gerais, Buenopolis (Not exact locality)	*T. sordida*	(2)
Hap_18	*T.sordida.s.s*	-18	-44	Brazil, Minas Gerais, Buenopolis (Not exact locality)	*T. sordida*	(2)
Hap_18	*T.sordida.s.s*	-13	-46	Brazil, Tocantins, Aurora de Tocantins		This paper
Hap_19	*T.sordida.s.s*	-17,87277778	-44,18	Brazil, Minas Gerais, Buenopolis (Not exact locality)	*T. sordida*	(2)
Hap_20	*T.sordida.s.s*	-19,75	-47,93	Brazil, Minas Gerais. Uberaba		This paper
Hap_21	*T.sordida.s.s*	-14,717401	-68,413711	Bolivia, La Paz, Apolo	*T. sordida*	Unpublished Belintani
Hap_21	*T.sordida.s.s*	-16,49416	-68,147	Bolivia, La Paz. Colony	*T. sordida*	(1,4)
Hap_22	*T.sordida.s.s*	-17,87277778	-44,18	Brazil, Minas Gerais, Buenopolis (Not exact locality)	*T. sordida*	(2)
Hap_23	*T.sordida.s.s*	-18	-44	Brazil, Minas Gerais, Buenopolis (Not exact locality)	*T. sordida*	(2)
Hap_24	*T.sordida.s.s*	-15,38	-56,1	Brazil, Mato Grosso, Varzea Grande		This paper
Hap_24	*T.sordida.s.s*	-18	-44	Brazil, Minas Gerais, Buenopolis (Not exact locality)	*T. sordida*	(2)
Hap_24	*T.sordida.s.s*	-17	-66	Bolivia, Cochabamba, chaco Tita. Colony. (Not exact coordinates)	*T. guasayana*	(1)
Hap_25	*T.sordida.s.s*	-17,87277778	-44,18	Brazil, Minas Gerais, Buenopolis (Not exact locality)	*T. sordida*	(2)
Hap_26	*T.sordida.s.s*	-18	-44	Brazil, Minas Gerais, Buenopolis (Not exact locality)	*T. sordida*	(2)
Hap_27	*T.sordida.s.s*	-12,51	-46,33	Brazil, Tocantins, Combinado		This paper
Hap_27	*T.sordida.s.s*	-15,38	-56	Brazil, Mato Grosso, Varzea Grande		This paper
Hap_28	*T.sordida.s.s*	-18	-44	Brazil, Minas Gerais, Buenopolis (Not exact locality)	*T. sordida*	(2)
Hap_29	*T.sordida.s.s*	-18	-44	Brazil, Minas Gerais, Buenopolis (Not exact locality)	*T. sordida*	(2)
Hap_30	*T.sordida.s.s*	-17,87277778	-44,18	Brazil, Minas Gerais, Buenopolis (Not exact locality)	*T. sordida*	(2)
Hap_31	*T.sordida.s.s*	-18	-44	Brazil, Minas Gerais, Buenopolis (Not exact locality)	*T. sordida*	(2)
Hap_32	*T.sordida.s.s*	-19,401315	-62,750258	Bolivia, Santa Cruz, Izozog.		This paper
Hap_32	*T.sordida.s.s*	-13	-46	Brazil, Tocantins, Aurora de Tocantins		This paper
Hap_32	*T.sordida.s.s*	-16,74	-43,86	Brazil, Minas Gerais, Montes Claros		This paper
Hap_32	*T.sordida.s.s*	-16,464419	-54,249643	Brazil, Mato Grosso, Sao Jose do Povo		This paper
Hap_32	*T.sordida.s.s*	-18	-44	Brazil, Minas Gerais, Buenopolis (Not exact locality)	*T. sordida*	(2)
Hap_32	*T.sordida.s.s*	-19	-58	Brazil, Mato Grosso do Sul, Corumba (Not an exact coordinates)	*T. sordida*	(4)
Hap_32	*T.sordida.s.s*	-12	-42	Brazil, Bahia, Seabra (Not an exact coordinates)	*T. sordida*	Unpublished Belintani
Hap_32	*T.sordida.s.s*			Brazil, Minas Gerais, Monte Azul. Colony.	*T. sordida*	Unpublished Belintani
Hap_32	*T.sordida.s.s*	-15,663889	-44,163889	Brazil, Minas Gerais, Ibiracatu. Colony.	*T. sordida*	(1)
Hap_32	*T.sordida.s.s*	-19	-57	Brazil, Mato Grosso do Sul, Pantanal. Colony. (Not exact locality)	*T. sordida*	(1)
Hap_32	*T.sordida.s.s*	-14	-46	Brazil, Goias, Posse. Colony.	*T. sordida*	(1)

Haplotype	Lineage	Latitud	Longitud	Geographic origin	NCBI species	Reference
Hap_33	*T.garciabesi*	-23	-64	Argentina. Salta. Hickman		This paper
Hap_34	*T.garciabesi*	-24,83	-60,03	Argentina, Formosa, Patiño.		This paper
Hap_35	*T.garciabesi*	-24,1833333	-62,88333	Argentina, Salta, Rivadavia. Colony. (Not exact coordinates)	*T. garciabesi*	(1)/Unpublished Belintani
Hap_35	*T.garciabesi*	-24,1833333	-62,88333	Argentina, Salta (Not exact locality)		This paper
Hap_36	*T.garciabesi*	-31,35	-66,62	Argentina, La Rioja, Rosario Vera Peñaloza		This paper
Hap_36	*T.garciabesi*	-28	-64	Argentina, Santiago del Estero (Not exact locality)		This paper
Hap_36	*T.garciabesi*	-32,38138	-68,05531	Argentina, Mendoza, Reserva Natural Bosques Telteca		This paper
Hap_37	*T.garciabesi*	-26	-61	Argentina, Salta, Balbuena		This paper
Hap_38	*T.garciabesi*	-24	-63	Argentina, Salta, Rivadavia		This paper
Hap_39	*T.garciabesi*	-19	-62,750258	Bolivia, Santa Cruz, Izozog		This paper
Hap_39	*T.garciabesi*	-17,796675	-63,080339	Bolivia, Santa Cruz. Colony.	*T. sordida*	(1)
Hap_40	*T.garciabesi*	-22	-60,95	Paraguay, Boqueron		This paper
Hap_41	*T.garciabesi*	-22	-60,95	Paraguay, Boqueron		This paper
Hap_42	*T.garciabesi*	-29	-63	Argentina, Santigo Estero, Aguirre		This paper
Hap_42	*T.garciabesi*	-28,985	-63,450556	Argentina, Santigo Estero, Salavina		This paper
Hap_43	*T.garciabesi*	-24,1833333	-62,88333	Argentina, Salta, Rivadavia		This paper
Hap_44	*T.sordida.s.l_1*	-28,985	-63,450556	Argentina, Santigo Estero, Salavina		This paper
Hap_45	*T.rosai*	-28	-57,587296	Argentina, Corrientes, San Miguel. Colony.	*T. sordida*	(1)
Hap_46	*T.rosai*	-23,914028	-56,701555	Paraguay, San Pedro, Nueva Germania		This paper
Hap_46	*T.rosai*	-28	-59	Argentina, Corrientes, San Luis del Palmar.		This paper
Hap_47	*T.rosai*	-25,533195	-57,079496	Paraguay, Paraguarí, Paraguarí, Naranjo		This paper
Hap_48	*T.rosai*	-28	-64	Argentina, Santiago del Estero (Not exact locality)		This paper
Hap_48	*T.rosai*	-28,229857	-61	Argentina, Santa Fe, El nochero		This paper
Hap_48	*T.rosai*	-26,96	-60,2	Argentina, Chaco, Guemes, Colonia Aborigen		This paper
Hap_48	*T.rosai*	-26	-60	Argentina, Chaco, Guemes, Paraje El Colchon		This paper
Hap_48	*T.rosai*	-26,804658	-59,997436	Argentina, Chaco, Veinticinco de Mayo, El Triangulo		This paper
Hap_48	*T.rosai*	-24,93945	-59,029187	Argentina, Formosa, Patiño, General Manuel Belgrano		This paper
Hap_48	*T.rosai*	-25,64	-60,93	Argentina, Chaco, Guemes, La Esperanza		This paper
Hap_48	*T.rosai*	-26	-60,26	Argentina, Chaco, Maipu, La Matanza		This paper
Hap_48	*T.rosai*	-32	-68	Argentina, Mendoza, Reserva Natural Bosques Telteca		This paper
Hap_48	*T.rosai*	-27,78	-64,26	Argentina, Santiago del Estero (Not exact locality)		This paper
Hap_48	*T.rosai*	-24	-57,083333	Paraguay, San Pedro, San Pedro, Yatebu Rugua		This paper
Hap_48	*T.rosai*	-26	-56,988926	Paraguay, Paraguarí, Escobar, Chircal		This paper
Hap_48	*T.rosai*	-24	-59,789786	Paraguay, Presidente Hayes, Teniente Esteban Martínez, Tte. Esteban Martínez		This paper
Hap_48	*T.rosai*	-26,4	-60,43	Argentina, Chaco, Maipu, Tres Isletas		This paper
Hap_48	*T.rosai*	-27	-58,83	Argentina, Corrientes (Not exact locality)		This paper
Hap_49	*T.rosai*	-26	-57	Paraguay, Paraguarí, Escobar, Chircal		This paper
Hap_49	*T.rosai*	-26	-57	Paraguay, Paraguarí, Paraguarí, Mbatovi		This paper
Hap_50	*T.sordida.La.Paz*	-14,717401	-68,413711	Bolivia, La Paz, Apolo	*T. guasayana*	Unpublished Belintani
Hap_51	*T.sordida.La.Paz*	-17	-67,136336	Bolivia, La Paz, Inquisivi		This paper
Hap_52	*T.sordida.La.Paz*	-17,796675	-63,080339	Bolivia, Santa Cruz. Colony. (Not exact locality)	*T. guasayana*	(1)
Hap_52	*T.sordida.La.Paz*	-17	-66	Bolivia, Cochabamba, chaco Tita. Colony. (Not exact coordinates)	*T. guasayana*	(1)
Hap_53	*T.sordida.s.l_2*	-17	-66	Bolivia, Cochabamba, Quillacollo, Cotapachi	*T. sordida*	(5)
Hap_54	*T.sordida.s.l_2*	-17	-66	Bolivia, Cochabamba (Not exact coordinates)	*T. sordida*	(6)
Hap_55	*T.sordida.s.l_2*	-23	-60	Paraguay, Boqueron, Tiberia		This paper
Hap_56	*T.sordida.s.l_2*	-17	-68	Bolivia, La Paz, Murillo, Mecapaca, Aucani	*T. sordida*	(5)

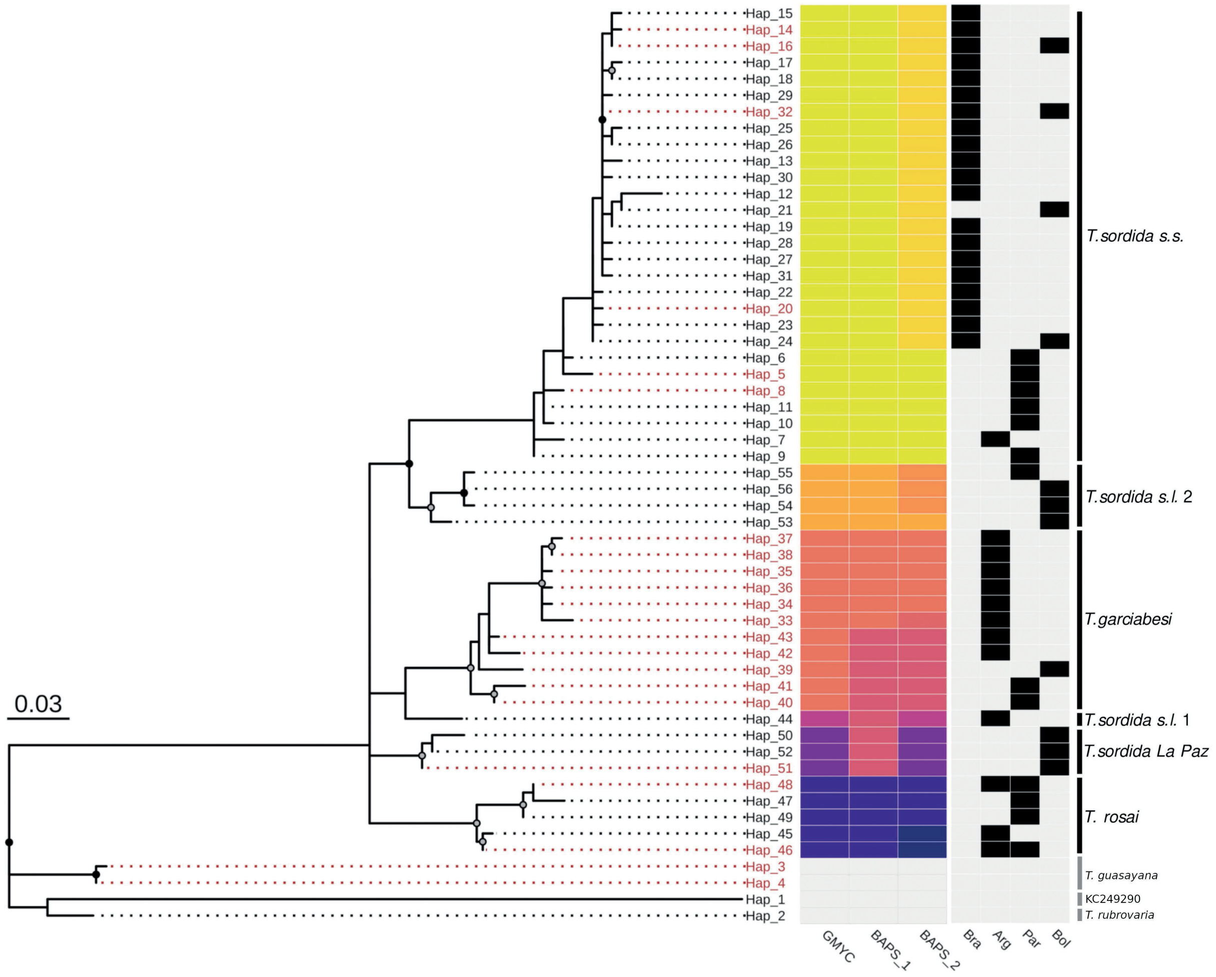

Fig. 1: maximum likelihood tree obtained from a *cytochrome b* fragment. Values of Bayesian posterior probabilities are depicted with numbers and circles on the nodes.

TABLE II

Mitochondrial haplotypes (*cytb*) and GenBank accession numbers of the sequences here analysed

Haplotype	Acc. Number
Hap_1	KC249290
Hap_2	KC249287
Hap_3	PP972075
Hap_4	PP972076
Hap_5	PP972083
Hap_6	PP972082
Hap_7	PP972084
Hap_8	PP972087
Hap_9	PP972088
Hap_10	PP972085
Hap_11	PP972086
Hap_12	PP972077
Hap_13	PP972080
Hap_14	PP972079
Hap_15	KR822194
Hap_16	KC608980
Hap_17	KR822187
Hap_18	KR822188
Hap_19	KR822193
Hap_20	PP972078
Hap_21	KC249291
Hap_21	MH054942
Hap_21	MZ700101
Hap_22	KR822190
Hap_23	KR822191
Hap_24	KC249250
Hap_24	KR822189
Hap_25	KR822198
Hap_26	KR822197
Hap_27	PP972081
Hap_28	KR822192
Hap_29	KR822195
Hap_30	KR822196
Hap_31	KR822199
Hap_32	MH054941
Hap_32	KC249289
Hap_32	KC249292
Hap_32	KC249294
Hap_32	MH054940
Hap_32	MZ700100
Hap_32	KR822185
Hap_33	PP972096
Hap_34	PP972100
Hap_35	KC249249
Hap_35	MH054943
Hap_36	PP972099
Hap_37	PP972097
Hap_38	PP972098
Hap_39	KC249293
Hap_40	PP972104
Hap_41	PP972103
Hap_42	PP972101
Hap_43	PP972102
Hap_44	PP972090
Hap_45	KC249295
Hap_46	PP972091
Hap_47	PP972092
Hap_48	PP972094
Hap_49	PP972093
Hap_50	MH054944
Hap_51	PP972095
Hap_52	KC249252
Hap_52	KC249253
Hap_53	HQ333243
Hap_54	AF045730
Hap_55	PP972089
Hap_56	HQ333242

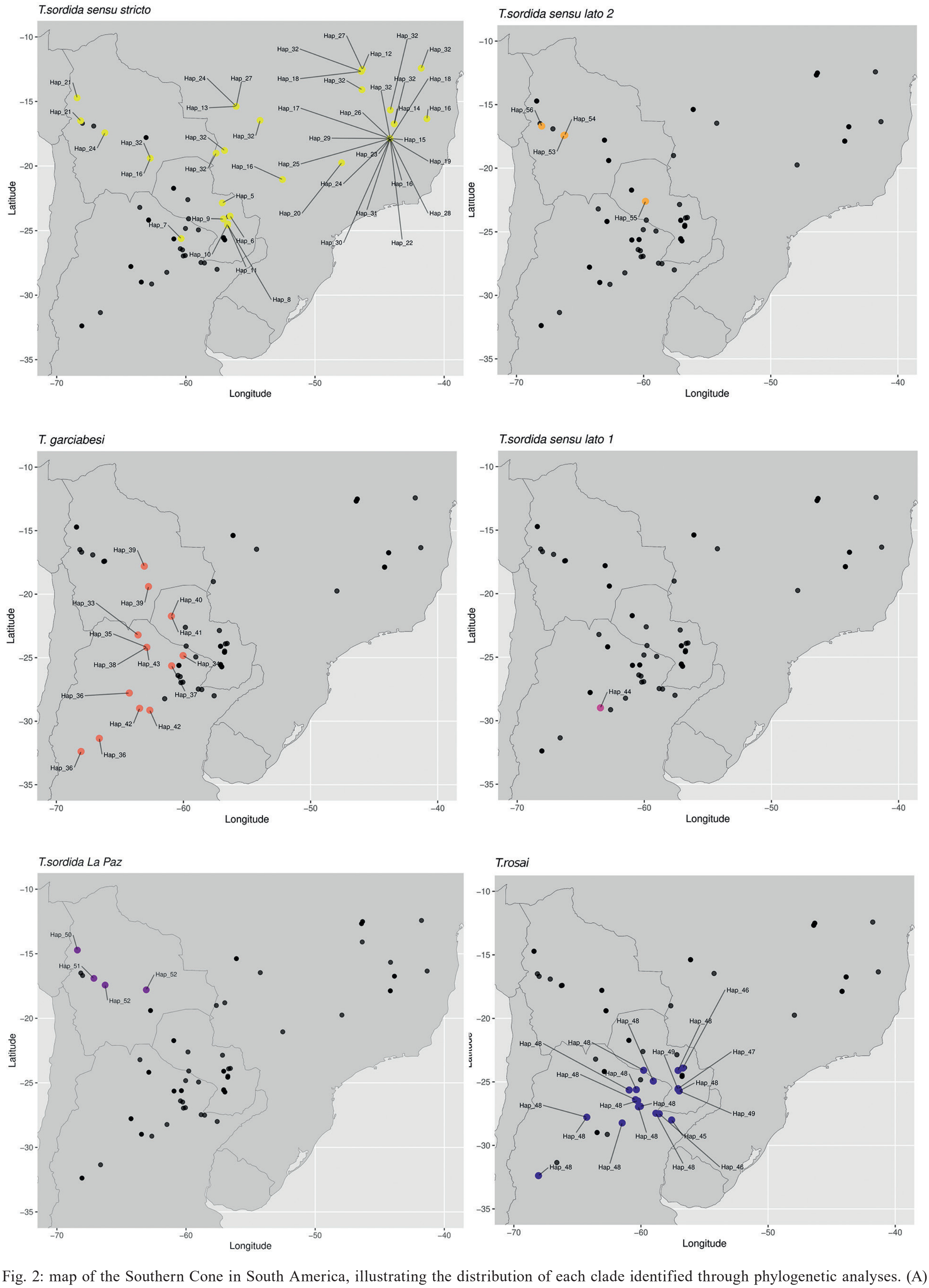

Fig. 2: map of the Southern Cone in South America, illustrating the distribution of each clade identified through phylogenetic analyses. (A) *Triatoma sordida sensu stricto*; (B) *T. sordida sensu lato* 2; (C) *T. garciabesi*; (D) *T. sordida sensu lato* 1; (E) *T. sordida* La Paz; (F) *T. rosai*.

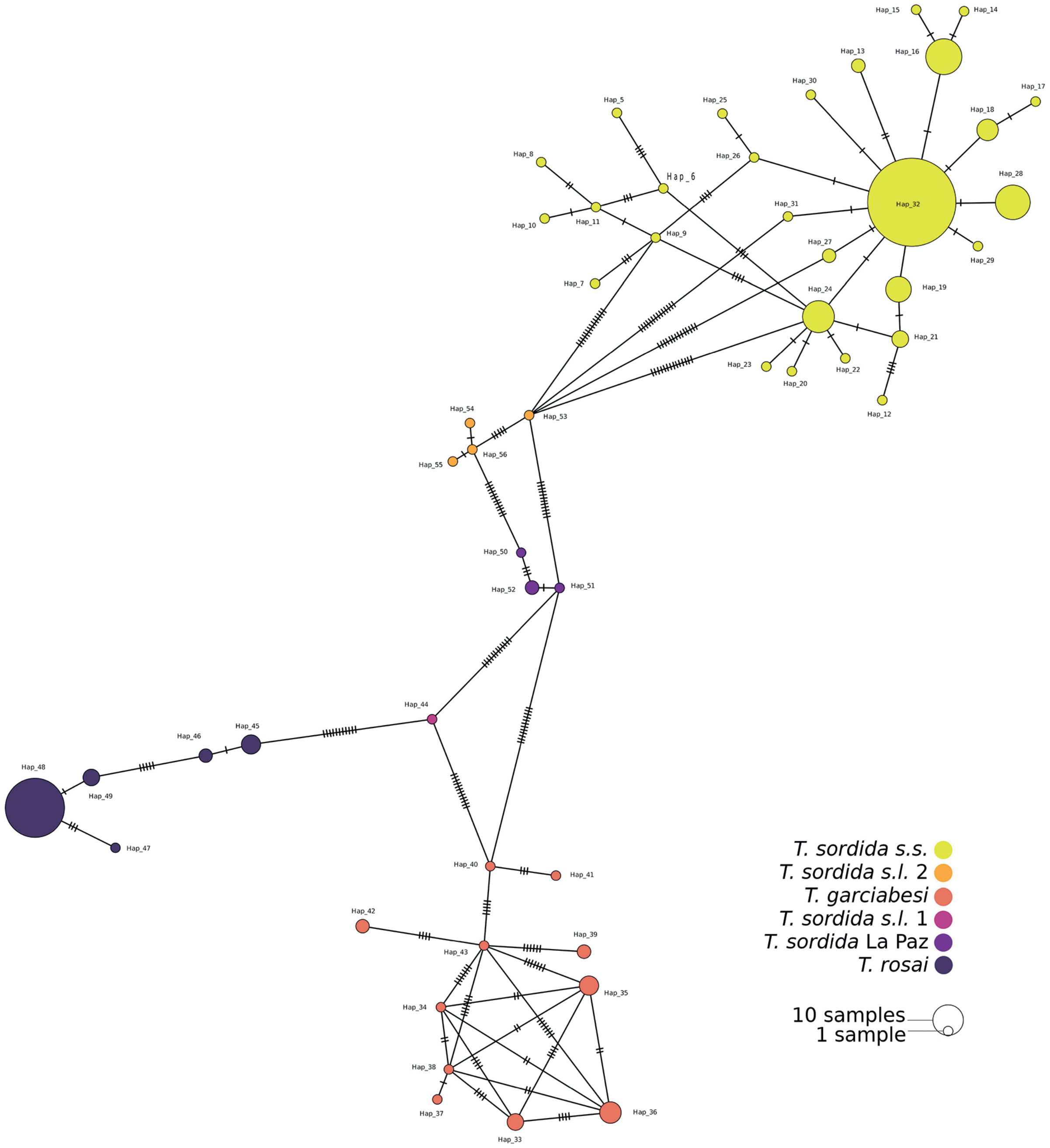

Fig. 3: minimum-spanning haplotype network based on a 233 bp *cytb* alignment. The nodes are haplotypes, with node size proportional to haplotype frequency. The numbers of mutational steps separating haplotypes are represented by dashes along the edges.

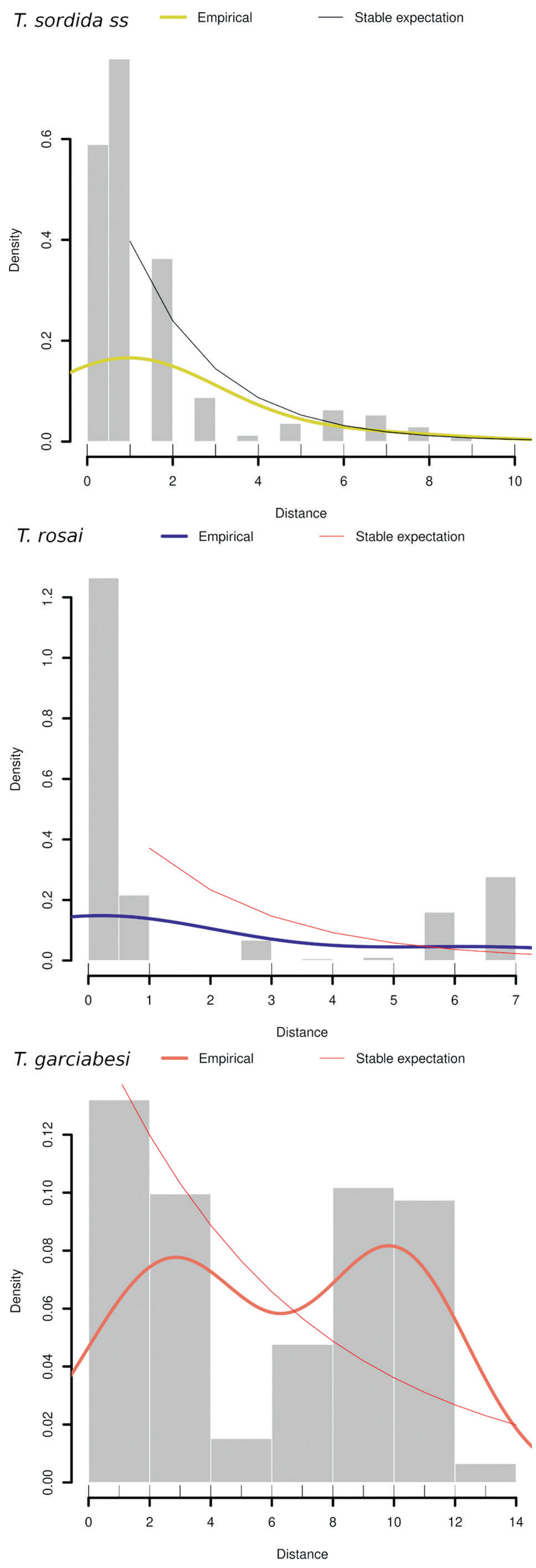

Fig. 4: mismatch distribution plot showing the relationship between genetic distance and abundance for *Triatoma sordida sensu stricto*, *T. rosai* and *T. garciabesi*.

TABLE III

Kimura 2-parameter (K2-p) genetic distances computed by haplotypes

	Hap_1	Hap_2	Hap_3	Hap_4	Hap_5	Hap_6	Hap_7	Hap_8	Hap_9	Hap_10	Hap_11	Hap_12	Hap_13	Hap_14	Hap_15	Hap_16	Hap_17	Hap_18	Hap_19	Hap_20	Hap_21	Hap_22	Hap_23	Hap_24	Hap_25	Hap_26	Hap_27	Hap_28	Hap_29	Hap_30	Hap_31	Hap_32	Hap_33	Hap_34	Hap_35	Hap_36	Hap_37	Hap_38	Hap_39	Hap_40	Hap_41	Hap_42	Hap_43	Hap_44	Hap_45	Hap_46	Hap_47	Hap_48	Hap_49	Hap_50	Hap_51	Hap_52	Hap_53	Hap_54	Hap_55	Hap_56
Hap_1	0	0,15988	0,17657	0,17131	0,24074	0,23433	0,22800	0,24648	0,22800	0,24074	0,23433	0,24074	0,25381	0,26048	0,25963	0,25381	0,25963	0,25381	0,24723	0,24723	0,24074	0,24723	0,24002	0,24074	0,25963	0,25381	0,24723	0,25301	0,25301	0,24074	0,24074	0,24723	0,23505	0,22868	0,23505	0,24150	0,23433	0,22868	0,22940	0,22307	0,22940	0,24150	0,24231	0,21065	0,22239	0,22868	0,22940	0,24150	0,23505	0,22175	0,22868	0,22239	0,23505	0,22868	0,22868	0,22239
Hap_2	0,15988	0	0,06861	0,06401	0,16666	0,14406	0,15523	0,16609	0,15523	0,16666	0,16091	0,15523	0,13856	0,14961	0,14914	0,14406	0,14914	0,14406	0,13856	0,13856	0,13313	0,13856	0,13815	0,13313	0,14914	0,14406	0,13313	0,14362	0,14362	0,13313	0,13313	0,13856	0,12211	0,13815	0,13815	0,13815	0,13778	0,13275	0,13856	0,14961	0,15523	0,14362	0,13313	0,12775	0,14914	0,14362	0,15523	0,15473	0,14914	0,13856	0,13901	0,14453	0,12814	0,14453	0,14453	0,13901
Hap_3	0,17657	0,06861	0	0,00431	0,17772	0,17772	0,17772	0,18896	0,17772	0,18963	0,18364	0,20182	0,19569	0,19569	0,19498	0,18963	0,20108	0,20182	0,19569	0,19569	0,18963	0,19569	0,19498	0,18963	0,20725	0,20182	0,18963	0,20108	0,20108	0,18963	0,18963	0,19569	0,13240	0,13778	0,13778	0,13778	0,13744	0,13240	0,13815	0,13815	0,14362	0,12243	0,13275	0,12740	0,16556	0,15988	0,16609	0,17131	0,17712	0,12211	0,12775	0,12243	0,14961	0,15523	0,15523	0,14961
Hap_4	0,17131	0,06401	0,00431	0	0,17247	0,17247	0,17247	0,18364	0,17247	0,18431	0,17836	0,19643	0,19033	0,19033	0,18963	0,18431	0,19569	0,19643	0,19033	0,19033	0,18431	0,19033	0,18963	0,18431	0,20182	0,19643	0,18431	0,19569	0,19569	0,18431	0,18431	0,19033	0,12740	0,13275	0,13275	0,13275	0,13240	0,12740	0,13313	0,13313	0,13856	0,12740	0,12775	0,12243	0,16038	0,15473	0,16091	0,16609	0,17187	0,11717	0,12279	0,11750	0,14453	0,15012	0,15012	0,14453
Hap_5	0,24074	0,16666	0,17772	0,17247	0	0,01747	0,03098	0,03086	0,02644	0,02644	0,02193	0,04020	0,04020	0,03098	0,03086	0,02644	0,04004	0,03557	0,03557	0,03098	0,03098	0,03098	0,03086	0,02644	0,04004	0,03557	0,03557	0,03543	0,03543	0,03557	0,03557	0,03098	0,11917	0,13004	0,11917	0,13004	0,12408	0,11917	0,11429	0,10897	0,12511	0,10329	0,10371	0,09850	0,09811	0,09298	0,09335	0,09811	0,09298	0,10371	0,09375	0,08862	0,08354	0,09375	0,09375	0,08862
Hap_6	0,23433	0,14406	0,17772	0,17247	0,01747	0	0,02193	0,02185	0,01747	0,01747	0,01304	0,03098	0,02193	0,03098	0,03086	0,02644	0,03086	0,02644	0,02644	0,02193	0,02193	0,02193	0,02185	0,01747	0,03086	0,02644	0,02644	0,02634	0,02634	0,02644	0,02644	0,02193	0,10329	0,11382	0,10329	0,11382	0,10812	0,10329	0,09850	0,09335	0,10897	0,09811	0,08825	0,08320	0,07791	0,07298	0,07325	0,07791	0,07298	0,09850	0,08354	0,08862	0,07354	0,08354	0,08354	0,07852
Hap_7	0,22800	0,15523	0,17772	0,17247	0,03098	0,02193	0	0,02634	0,01304	0,02193	0,01747	0,03557	0,03557	0,03557	0,03543	0,03098	0,03543	0,03098	0,03098	0,02644	0,02644	0,02644	0,02634	0,02193	0,02634	0,02193	0,03098	0,03086	0,03086	0,03098	0,03098	0,02644	0,10329	0,11382	0,10329	0,11382	0,10812	0,10329	0,09850	0,09335	0,10897	0,09811	0,08825	0,08320	0,09298	0,08791	0,07820	0,09298	0,08791	0,11429	0,09893	0,10416	0,07852	0,08862	0,08862	0,08354
Hap_8	0,24648	0,16609	0,18896	0,18364	0,03086	0,02185	0,02634	0	0,01300	0,01300	0,00863	0,04469	0,04469	0,04469	0,04453	0,04004	0,04453	0,04004	0,04004	0,03543	0,03543	0,03543	0,03531	0,03086	0,03531	0,03086	0,04004	0,03990	0,03990	0,04004	0,04004	0,03543	0,10812	0,11870	0,10812	0,11870	0,11297	0,10812	0,10329	0,10329	0,11917	0,10291	0,10329	0,09298	0,09265	0,08760	0,08791	0,09265	0,08760	0,11382	0,09850	0,10371	0,07820	0,08825	0,08825	0,08320
Hap_9	0,22800	0,15523	0,17772	0,17247	0,02644	0,01747	0,01304	0,01300	0	0,00866	0,00431	0,03098	0,03098	0,03098	0,03086	0,02644	0,03086	0,02644	0,02644	0,02193	0,02193	0,02193	0,02185	0,01747	0,02185	0,01747	0,02644	0,02634	0,02634	0,02644	0,02644	0,02193	0,09811	0,10853	0,09811	0,10853	0,10291	0,09811	0,09335	0,08825	0,10371	0,09298	0,09335	0,07820	0,07791	0,07298	0,07325	0,07791	0,07298	0,09850	0,08354	0,08862	0,06374	0,07354	0,07354	0,06861
Hap_10	0,24074	0,16666	0,18963	0,18431	0,02644	0,01747	0,02193	0,01300	0,00866	0	0,00431	0,04020	0,04020	0,04020	0,04004	0,03557	0,04004	0,03557	0,03557	0,03098	0,03098	0,03098	0,03086	0,02644	0,03086	0,02644	0,03557	0,03543	0,03543	0,03557	0,03557	0,03098	0,10853	0,11917	0,10853	0,11917	0,11338	0,10853	0,10371	0,09850	0,11429	0,10329	0,10371	0,08825	0,08791	0,08288	0,08320	0,08791	0,08288	0,10897	0,09375	0,09893	0,07354	0,08354	0,08354	0,07852
Hap_11	0,23433	0,16091	0,18364	0,17836	0,02193	0,01304	0,01747	0,00863	0,00431	0,00431	0	0,03557	0,03557	0,03557	0,03543	0,03098	0,03543	0,03098	0,03098	0,02644	0,02644	0,02644	0,02634	0,02193	0,02634	0,02193	0,03098	0,03086	0,03086	0,03098	0,03098	0,02644	0,10329	0,11382	0,10329	0,11382	0,10812	0,10329	0,09850	0,09335	0,10897	0,09811	0,09850	0,08320	0,08288	0,07791	0,07820	0,08288	0,07791	0,10371	0,08862	0,09375	0,06861	0,07852	0,07852	0,07354
Hap_12	0,24074	0,15523	0,20182	0,19643	0,04020	0,03098	0,03557	0,04469	0,03098	0,04020	0,03557	0	0,03557	0,03557	0,03543	0,03098	0,03543	0,03098	0,02193	0,02644	0,01747	0,02644	0,02634	0,02193	0,03543	0,03098	0,03098	0,03086	0,03086	0,03098	0,03098	0,02644	0,10329	0,12457	0,11382	0,12457	0,11870	0,11382	0,10897	0,10371	0,11967	0,10853	0,09850	0,10371	0,10329	0,09811	0,09850	0,10329	0,09811	0,11429	0,10945	0,11480	0,08862	0,07852	0,07852	0,07354
Hap_13	0,25381	0,13856	0,19569	0,19033	0,04020	0,02193	0,03557	0,04469	0,03098	0,04020	0,03557	0,03557	0	0,01747	0,01741	0,01304	0,01741	0,01304	0,01304	0,01747	0,01747	0,01747	0,01741	0,01304	0,01741	0,01304	0,01304	0,01300	0,01300	0,01304	0,01304	0,00866	0,08791	0,10853	0,09811	0,10853	0,10291	0,09811	0,08320	0,08825	0,10371	0,09298	0,08320	0,08825	0,07298	0,06810	0,06834	0,07298	0,06810	0,09335	0,07852	0,08354	0,06861	0,08862	0,08862	0,08354
Hap_14	0,26048	0,14961	0,19569	0,19033	0,03098	0,03098	0,03557	0,04469	0,03098	0,04020	0,03557	0,03557	0,01747	0	0,00863	0,00431	0,01741	0,01304	0,01304	0,01747	0,01747	0,01747	0,01741	0,01304	0,01741	0,01304	0,01304	0,01300	0,01300	0,01304	0,01304	0,00866	0,10329	0,12457	0,11382	0,12457	0,11870	0,11382	0,09850	0,10371	0,11967	0,09811	0,09850	0,10371	0,09298	0,08791	0,08825	0,09298	0,08791	0,10897	0,08862	0,09375	0,07852	0,09893	0,09893	0,09375
Hap_15	0,25963	0,14914	0,19498	0,18963	0,03086	0,03086	0,03543	0,04453	0,03086	0,04004	0,03543	0,03543	0,01741	0,00863	0	0,00431	0,01737	0,01300	0,01300	0,01741	0,01741	0,01741	0,01737	0,01300	0,00866	0,01300	0,01300	0,01299	0,01299	0,01300	0,01300	0,00863	0,10291	0,12408	0,11338	0,12408	0,11826	0,11338	0,09811	0,10329	0,11917	0,09775	0,09811	0,10329	0,09265	0,08760	0,08791	0,09265	0,08760	0,10853	0,08825	0,09335	0,07820	0,09850	0,09850	0,09335
Hap_16	0,25381	0,14406	0,18963	0,18431	0,02644	0,02644	0,03098	0,04004	0,02644	0,03557	0,03098	0,03098	0,01304	0,00431	0,00431	0	0,01300	0,00866	0,00866	0,01304	0,01304	0,01304	0,01300	0,00866	0,01300	0,00866	0,00866	0,00863	0,00863	0,00866	0,00866	0,00431	0,09811	0,11917	0,10853	0,11917	0,11338	0,10853	0,09335	0,09850	0,11429	0,09298	0,09335	0,09850	0,08791	0,08288	0,08320	0,08791	0,08288	0,10371	0,08354	0,08862	0,07354	0,09375	0,09375	0,08862
Hap_17	0,25963	0,14914	0,20108	0,19569	0,04004	0,03086	0,03543	0,04453	0,03086	0,04004	0,03543	0,03543	0,01741	0,01741	0,01737	0,01300	0	0,00431	0,01300	0,01741	0,01741	0,01741	0,01737	0,01300	0,01737	0,01300	0,01300	0,01299	0,01299	0,01300	0,01300	0,00863	0,10291	0,12408	0,11338	0,11338	0,11826	0,11338	0,09811	0,10329	0,11917	0,10812	0,09811	0,10329	0,09265	0,08760	0,08288	0,08760	0,08760	0,11382	0,09850	0,10371	0,07820	0,09850	0,09850	0,09335
Hap_18	0,25381	0,14406	0,20182	0,19643	0,03557	0,02644	0,03098	0,04004	0,02644	0,03557	0,03098	0,03098	0,01304	0,01304	0,01300	0,00866	0,00431	0	0,00866	0,01304	0,01304	0,01304	0,01300	0,00866	0,01300	0,00866	0,00866	0,00863	0,00863	0,00866	0,00866	0,00431	0,09811	0,11917	0,10853	0,10853	0,11338	0,10853	0,09335	0,09850	0,11429	0,10329	0,09335	0,09850	0,08791	0,08288	0,08320	0,08791	0,08288	0,10897	0,09375	0,09893	0,07354	0,09375	0,09375	0,08862
Hap_19	0,24723	0,13856	0,19569	0,19033	0,03557	0,02644	0,03098	0,04004	0,02644	0,03557	0,03098	0,02193	0,01304	0,01304	0,01300	0,00866	0,01300	0,00866	0	0,01304	0,00431	0,01304	0,01300	0,00866	0,01300	0,00866	0,00866	0,00863	0,00863	0,00866	0,00866	0,00431	0,08791	0,10853	0,09811	0,10853	0,10291	0,09811	0,08320	0,08825	0,10371	0,09298	0,08320	0,09850	0,08791	0,08288	0,08320	0,08791	0,08288	0,09850	0,09375	0,09893	0,07354	0,08354	0,08354	0,07852
Hap_20	0,24723	0,13856	0,19569	0,19033	0,03098	0,02193	0,02644	0,03543	0,02193	0,03098	0,02644	0,02644	0,01747	0,01747	0,01741	0,01304	0,01741	0,01304	0,01304	0	0,00866	0,00866	0,00863	0,00431	0,01741	0,01304	0,01304	0,01300	0,01300	0,01304	0,01304	0,00866	0,10329	0,12457	0,11382	0,12457	0,11870	0,11382	0,09850	0,10371	0,11967	0,10853	0,09850	0,09335	0,08288	0,07791	0,07820	0,08288	0,07791	0,11429	0,09893	0,10416	0,06861	0,08862	0,08862	0,08354
Hap_21	0,24074	0,13313	0,18963	0,18431	0,03098	0,02193	0,02644	0,03543	0,02193	0,03098	0,02644	0,01747	0,01747	0,01747	0,01741	0,01304	0,01741	0,01304	0,00431	0,00866	0	0,00866	0,00863	0,00431	0,01741	0,01304	0,01304	0,01300	0,01300	0,01304	0,01304	0,00866	0,09298	0,11382	0,10329	0,11382	0,10812	0,10329	0,08825	0,09335	0,10897	0,09811	0,08825	0,09335	0,08288	0,07791	0,07820	0,08288	0,07791	0,10371	0,09893	0,10416	0,06861	0,07852	0,07852	0,07354
Hap_22	0,24723	0,13856	0,19569	0,19033	0,03098	0,02193	0,02644	0,03543	0,02193	0,03098	0,02644	0,02644	0,01747	0,01747	0,01741	0,01304	0,01741	0,01304	0,01304	0,00866	0,00866	0	0,00863	0,00431	0,01741	0,01304	0,01304	0,01300	0,01300	0,01304	0,01304	0,00866	0,10329	0,12457	0,11382	0,12457	0,11870	0,11382	0,09850	0,10371	0,11967	0,10853	0,09850	0,09335	0,08288	0,07791	0,07820	0,08288	0,07791	0,11429	0,09893	0,10416	0,06861	0,08862	0,08862	0,08354
Hap_23	0,24002	0,13815	0,19498	0,18963	0,03086	0,02185	0,02634	0,03531	0,02185	0,03086	0,02634	0,02634	0,01741	0,01741	0,01737	0,01300	0,01737	0,01300	0,01300	0,00863	0,00863	0,00863	0	0,00431	0,01737	0,01300	0,01300	0,01299	0,01299	0,01300	0,01300	0,00863	0,10291	0,11870	0,11338	0,12408	0,11826	0,11338	0,09811	0,10329	0,11917	0,10812	0,09811	0,09298	0,08260	0,07765	0,07791	0,08260	0,07765	0,10853	0,09335	0,09850	0,06834	0,08825	0,08825	0,08320
Hap_24	0,24074	0,13313	0,18963	0,18431	0,02644	0,01747	0,02193	0,03086	0,01747	0,02644	0,02193	0,02193	0,01304	0,01304	0,01300	0,00866	0,01300	0,00866	0,00866	0,00431	0,00431	0,00431	0,00431	0	0,01300	0,00866	0,00866	0,00863	0,00863	0,00866	0,00866	0,00431	0,09811	0,11917	0,10853	0,11917	0,11338	0,10853	0,09335	0,09850	0,11429	0,10329	0,09335	0,08825	0,07791	0,07298	0,07325	0,07791	0,07298	0,10897	0,09375	0,09893	0,06374	0,08354	0,08354	0,07852
Hap_25	0,25963	0,14914	0,20725	0,20182	0,04004	0,03086	0,02634	0,03531	0,02185	0,03086	0,02634	0,03543	0,01741	0,01741	0,00866	0,01300	0,01737	0,01300	0,01300	0,01741	0,01741	0,01741	0,01737	0,01300	0	0,00431	0,01300	0,01299	0,01299	0,01300	0,01300	0,00863	0,10291	0,12408	0,11338	0,12408	0,11826	0,11338	0,09811	0,10329	0,11917	0,10812	0,09811	0,10329	0,09265	0,08760	0,08791	0,09265	0,08760	0,11382	0,09850	0,10371	0,07820	0,09850	0,09850	0,09335
Hap_26	0,25381	0,14406	0,20182	0,19643	0,03557	0,02644	0,02193	0,03086	0,01747	0,02644	0,02193	0,03098	0,01304	0,01304	0,01300	0,00866	0,01300	0,00866	0,00866	0,01304	0,01304	0,01304	0,01300	0,00866	0,00431	0	0,00866	0,00863	0,00863	0,00866	0,00866	0,00431	0,09811	0,11917	0,10853	0,11917	0,11338	0,10853	0,09335	0,09850	0,11429	0,10329	0,09335	0,09850	0,08791	0,08288	0,08320	0,08791	0,08288	0,10897	0,09375	0,09893	0,07354	0,09375	0,09375	0,08862
Hap_27	0,24723	0,13313	0,18963	0,18431	0,03557	0,02644	0,03098	0,04004	0,02644	0,03557	0,03098	0,03098	0,01304	0,01304	0,01300	0,00866	0,01300	0,00866	0,00866	0,01304	0,01304	0,01304	0,01300	0,00866	0,01300	0,00866	0	0,00863	0,00863	0,00866	0,00866	0,00431	0,08791	0,10853	0,09811	0,10853	0,10291	0,09811	0,09335	0,09850	0,11429	0,10329	0,09335	0,09850	0,08791	0,08288	0,08320	0,08791	0,08288	0,09850	0,09375	0,09893	0,06374	0,08354	0,08354	0,07852
Hap_28	0,25301	0,14362	0,20108	0,19569	0,03543	0,02634	0,03086	0,03990	0,02634	0,03543	0,03086	0,03086	0,01300	0,01300	0,01299	0,00863	0,01299	0,00863	0,00863	0,01300	0,01300	0,01300	0,01299	0,00863	0,01299	0,00863	0,00863	0	0,00864	0,00863	0,00863	0,00431	0,09775	0,11870	0,10812	0,11870	0,11297	0,10812	0,09298	0,09811	0,11382	0,10291	0,09298	0,09811	0,08760	0,08260	0,08288	0,08760	0,08260	0,10853	0,09335	0,09850	0,07325	0,09335	0,09335	0,08825
Hap_29	0,25301	0,14362	0,20108	0,19569	0,03543	0,02634	0,03086	0,03990	0,02634	0,03543	0,03086	0,03086	0,01300	0,01300	0,01299	0,00863	0,01299	0,00863	0,00863	0,01300	0,01300	0,01300	0,01299	0,00863	0,01299	0,00863	0,00863	0,00864	0	0,00863	0,00863	0,00431	0,09775	0,11870	0,10812	0,11870	0,11297	0,10812	0,09298	0,09811	0,11382	0,10291	0,09298	0,09811	0,08760	0,08260	0,08288	0,08760	0,08260	0,10853	0,09335	0,09850	0,07325	0,09335	0,09335	0,08825
Hap_30	0,24074	0,13313	0,18963	0,18431	0,03557	0,02644	0,03098	0,04004	0,02644	0,03557	0,03098	0,03098	0,01304	0,01304	0,01300	0,00866	0,01300	0,00866	0,00866	0,01304	0,01304	0,01304	0,01300	0,00866	0,01300	0,00866	0,00866	0,00863	0,00863	0	0,00866	0,00431	0,08791	0,10853	0,09811	0,10853	0,10291	0,09811	0,09335	0,09850	0,11429	0,10329	0,09335	0,09850	0,08791	0,08288	0,08320	0,08791	0,08288	0,10897	0,09375	0,09893	0,07354	0,09375	0,09375	0,08862
Hap_31	0,24074	0,13313	0,18963	0,18431	0,03557	0,02644	0,03098	0,04004	0,02644	0,03557	0,03098	0,03098	0,01304	0,01304	0,01300	0,00866	0,01300	0,00866	0,00866	0,01304	0,01304	0,01304	0,01300	0,00866	0,01300	0,00866	0,00866	0,00863	0,00863	0,00866	0	0,00431	0,09811	0,11917	0,10853	0,11917	0,11338	0,10853	0,09335	0,09850	0,11429	0,10329	0,09335	0,08825	0,07791	0,07298	0,07325	0,07791	0,07298	0,09850	0,08354	0,08862	0,06374	0,08354	0,08354	0,07852
Hap_32	0,24723	0,13856	0,19569	0,19033	0,03098	0,02193	0,02644	0,03543	0,02193	0,03098	0,02644	0,02644	0,00866	0,00866	0,00863	0,00431	0,00863	0,00431	0,00431	0,00866	0,00866	0,00866	0,00863	0,00431	0,00863	0,00431	0,00431	0,00431	0,00431	0,00431	0,00431	0	0,09298	0,11382	0,10329	0,11382	0,10812	0,10329	0,08825	0,09335	0,10897	0,09811	0,08825	0,09335	0,08288	0,07791	0,07820	0,08288	0,07791	0,10371	0,08862	0,09375	0,06861	0,08862	0,08862	0,08354
Hap_33	0,23505	0,12211	0,13240	0,12740	0,11917	0,10329	0,10329	0,10812	0,09811	0,10853	0,10329	0,10329	0,08791	0,10329	0,10291	0,09811	0,10291	0,09811	0,08791	0,10329	0,09298	0,10329	0,10291	0,09811	0,10291	0,09811	0,08791	0,09775	0,09775	0,08791	0,09811	0,09298	0	0,01747	0,01747	0,01747	0,02185	0,01747	0,04938	0,04469	0,05891	0,04920	0,04004	0,06861	0,10812	0,10291	0,09811	0,10812	0,10291	0,07791	0,08320	0,08825	0,08825	0,08825	0,08825	0,08320
Hap_34	0,22868	0,13815	0,13778	0,13275	0,13004	0,11382	0,11382	0,11870	0,10853	0,11917	0,11382	0,12457	0,10853	0,12457	0,12408	0,11917	0,12408	0,11917	0,10853	0,12457	0,11382	0,12457	0,11870	0,11917	0,12408	0,11917	0,10853	0,11870	0,11870	0,10853	0,11917	0,11382	0,01747	0	0,00866	0,00866	0,01300	0,00866	0,04938	0,04469	0,04938	0,03990	0,03086	0,07852	0,11870	0,11338	0,10853	0,10812	0,10291	0,06810	0,07325	0,07820	0,08825	0,08825	0,08825	0,08320
Hap_35	0,23505	0,13815	0,13778	0,13275	0,11917	0,10329	0,10329	0,10812	0,09811	0,10853	0,10329	0,11382	0,09811	0,11382	0,11338	0,10853	0,11338	0,10853	0,09811	0,11382	0,10329	0,11382	0,11338	0,10853	0,11338	0,10853	0,09811	0,10812	0,10812	0,09811	0,10853	0,10329	0,01747	0,00866	0	0,00866	0,01300	0,00866	0,04938	0,04469	0,04938	0,03990	0,03086	0,07852	0,11338	0,10812	0,10329	0,10291	0,09775	0,07791	0,08320	0,08825	0,07820	0,07820	0,07820	0,07325
Hap_36	0,24150	0,13815	0,13778	0,13275	0,13004	0,11382	0,11382	0,11870	0,10853	0,11917	0,11382	0,12457	0,10853	0,12457	0,12408	0,11917	0,11338	0,10853	0,10853	0,12457	0,11382	0,12457	0,12408	0,11917	0,12408	0,11917	0,10853	0,11870	0,11870	0,10853	0,11917	0,11382	0,01747	0,00866	0,00866	0	0,01300	0,00866	0,04938	0,04469	0,04938	0,03990	0,03086	0,07852	0,11870	0,11338	0,10853	0,10812	0,10291	0,07791	0,08320	0,08825	0,08825	0,08825	0,08825	0,08320
Hap_37	0,23433	0,13778	0,13744	0,13240	0,12408	0,10812	0,10812	0,11297	0,10291	0,11338	0,10812	0,11870	0,10291	0,11870	0,11826	0,11338	0,11826	0,11338	0,10291	0,11870	0,10812	0,11870	0,11826	0,11338	0,11826	0,11338	0,10291	0,11297	0,11297	0,10291	0,11338	0,10812	0,02185	0,01300	0,01300	0,01300	0	0,00431	0,04453	0,04920	0,05392	0,04440	0,03531	0,08320	0,11297	0,10774	0,10291	0,10256	0,09743	0,07275	0,07791	0,08288	0,08288	0,08288	0,08288	0,07791
Hap_38	0,22868	0,13275	0,13240	0,12740	0,11917	0,10329	0,10329	0,10812	0,09811	0,10853	0,10329	0,11382	0,09811	0,11382	0,11338	0,10853	0,11338	0,10853	0,09811	0,11382	0,10329	0,11382	0,11338	0,10853	0,11338	0,10853	0,09811	0,10812	0,10812	0,09811	0,10853	0,10329	0,01747	0,00866	0,00866	0,00866	0,00431	0	0,04004	0,04469	0,04938	0,03990	0,03086	0,07852	0,10812	0,10291	0,09811	0,09775	0,09265	0,06810	0,07325	0,07820	0,07820	0,07820	0,07820	0,07325
Hap_39	0,22940	0,13856	0,13815	0,13313	0,11429	0,09850	0,09850	0,10329	0,09335	0,10371	0,09850	0,10897	0,08320	0,09850	0,09811	0,09335	0,09811	0,09335	0,08320	0,09850	0,08825	0,09850	0,09811	0,09335	0,09811	0,09335	0,09335	0,09298	0,09298	0,09335	0,09335	0,08825	0,04938	0,04938	0,04938	0,04938	0,04453	0,04004	0	0,03098	0,04487	0,03543	0,02644	0,06892	0,08791	0,08288	0,08825	0,08791	0,08288	0,06349	0,05891	0,06374	0,06374	0,07354	0,07354	0,06861
Hap_40	0,22307	0,14961	0,13815	0,13313	0,10897	0,09335	0,09335	0,10329	0,08825	0,09850	0,09335	0,10371	0,08825	0,10371	0,10329	0,09850	0,10329	0,09850	0,08825	0,10371	0,09335	0,10371	0,10329	0,09850	0,10329	0,09850	0,09850	0,09811	0,09811	0,09850	0,09850	0,09335	0,04469	0,04469	0,04469	0,04469	0,04920	0,04469	0,03098	0	0,01304	0,03086	0,02193	0,05435	0,09298	0,08791	0,09335	0,09298	0,08791	0,05868	0,05412	0,05891	0,06861	0,06861	0,06861	0,06374
Hap_41	0,22940	0,15523	0,14362	0,13856	0,12511	0,10897	0,10897	0,11917	0,10371	0,11429	0,10897	0,11967	0,10371	0,11967	0,11917	0,11429	0,11917	0,11429	0,10371	0,11967	0,10897	0,11967	0,11917	0,11429	0,11917	0,11429	0,11429	0,11382	0,11382	0,11429	0,11429	0,10897	0,05891	0,04938	0,04938	0,04938	0,05392	0,04938	0,04487	0,01304	0	0,03543	0,02644	0,05916	0,09811	0,09298	0,10897	0,10853	0,10329	0,06349	0,05891	0,06374	0,08354	0,08354	0,08354	0,07852
Hap_42	0,24150	0,14362	0,12243	0,12740	0,10329	0,09811	0,09811	0,10291	0,09298	0,10329	0,09811	0,10853	0,09298	0,09811	0,09775	0,09298	0,10812	0,10329	0,09298	0,10853	0,09811	0,10853	0,10812	0,10329	0,10812	0,10329	0,10329	0,10291	0,10291	0,10329	0,10329	0,09811	0,04920	0,03990	0,03990	0,03990	0,04440	0,03990	0,03543	0,03086	0,03543	0	0,01741	0,05891	0,10291	0,09775	0,09811	0,10291	0,09775	0,06810	0,05868	0,06349	0,07325	0,07325	0,07325	0,06834
Hap_43	0,24231	0,13313	0,13275	0,12775	0,10371	0,08825	0,08825	0,10329	0,09335	0,10371	0,09850	0,09850	0,08320	0,09850	0,09811	0,09335	0,09811	0,09335	0,08320	0,09850	0,08825	0,09850	0,09811	0,09335	0,09811	0,09335	0,09335	0,09298	0,09298	0,09335	0,09335	0,08825	0,04004	0,03086	0,03086	0,03086	0,03531	0,03086	0,02644	0,02193	0,02644	0,01741	0	0,05916	0,09811	0,09298	0,09850	0,09811	0,09298	0,06349	0,05891	0,06374	0,07354	0,07354	0,07354	0,06861
Hap_44	0,21065	0,12775	0,12740	0,12243	0,09850	0,08320	0,08320	0,09298	0,07820	0,08825	0,08320	0,10371	0,08825	0,10371	0,10329	0,09850	0,10329	0,09850	0,09850	0,09335	0,09335	0,09335	0,09298	0,08825	0,10329	0,09850	0,09850	0,09811	0,09811	0,09850	0,08825	0,09335	0,06861	0,07852	0,07852	0,07852	0,08320	0,07852	0,06892	0,05435	0,05916	0,05891	0,05916	0	0,04905	0,05375	0,06349	0,07791	0,07298	0,06834	0,05412	0,05891	0,05891	0,06861	0,06861	0,06374
Hap_45	0,22239	0,14914	0,16556	0,16038	0,09811	0,07791	0,09298	0,09265	0,07791	0,08791	0,08288	0,10329	0,07298	0,09298	0,09265	0,08791	0,09265	0,08791	0,08791	0,08288	0,08288	0,08288	0,08260	0,07791	0,09265	0,08791	0,08791	0,08760	0,08760	0,08791	0,07791	0,08288	0,10812	0,11870	0,11338	0,11870	0,11297	0,10812	0,08791	0,09298	0,09811	0,10291	0,09811	0,04905	0	0,00431	0,03086	0,03098	0,02644	0,07791	0,06349	0,06834	0,06349	0,08320	0,08320	0,07820
Hap_46	0,22868	0,14362	0,15988	0,15473	0,09298	0,07298	0,08791	0,08760	0,07298	0,08288	0,07791	0,09811	0,06810	0,08791	0,08760	0,08288	0,08760	0,08288	0,08288	0,07791	0,07791	0,07791	0,07765	0,07298	0,08760	0,08288	0,08288	0,08260	0,08260	0,08288	0,07298	0,07791	0,10291	0,11338	0,10812	0,11338	0,10774	0,10291	0,08288	0,08791	0,09298	0,09775	0,09298	0,05375	0,00431	0	0,02634	0,02644	0,02193	0,07298	0,05868	0,06349	0,05868	0,07820	0,07820	0,07325
Hap_47	0,22940	0,15523	0,16609	0,16091	0,09335	0,07325	0,07820	0,08791	0,07325	0,08320	0,07820	0,09850	0,06834	0,08825	0,08791	0,08320	0,08288	0,08320	0,08320	0,07820	0,07820	0,07820	0,07791	0,07325	0,08791	0,08320	0,08320	0,08288	0,08288	0,08320	0,07325	0,07820	0,09811	0,10853	0,10329	0,10853	0,10291	0,09811	0,08825	0,09335	0,10897	0,09811	0,09850	0,06349	0,03086	0,02634	0	0,01300	0,01741	0,09850	0,08354	0,08862	0,06861	0,07852	0,07852	0,07354
Hap_48	0,24150	0,15473	0,17131	0,16609	0,09811	0,07791	0,09298	0,09265	0,07791	0,08791	0,08288	0,10329	0,07298	0,09298	0,09265	0,08791	0,08760	0,08791	0,08791	0,08288	0,08288	0,08288	0,08260	0,07791	0,09265	0,08791	0,08791	0,08760	0,08760	0,08791	0,07791	0,08288	0,10812	0,10812	0,10291	0,10812	0,10256	0,09775	0,08791	0,09298	0,10853	0,10291	0,09811	0,07791	0,03098	0,02644	0,01300	0	0,00431	0,08791	0,07325	0,07820	0,06349	0,07325	0,07325	0,06834
Hap_49	0,23505	0,14914	0,17712	0,17187	0,09298	0,07298	0,08791	0,08760	0,07298	0,08288	0,07791	0,09811	0,06810	0,08791	0,08760	0,08288	0,08760	0,08288	0,08288	0,07791	0,07791	0,07791	0,07765	0,07298	0,08760	0,08288	0,08288	0,08260	0,08260	0,08288	0,07298	0,07791	0,10291	0,10291	0,09775	0,10291	0,09743	0,09265	0,08288	0,08791	0,10329	0,09775	0,09298	0,07298	0,02644	0,02193	0,01741	0,00431	0	0,08288	0,06834	0,07325	0,05868	0,06834	0,06834	0,06349
Hap_50	0,22175	0,13856	0,12211	0,11717	0,10371	0,09850	0,11429	0,11382	0,09850	0,10897	0,10371	0,11429	0,09335	0,10897	0,10853	0,10371	0,11382	0,10897	0,09850	0,11429	0,10371	0,11429	0,10853	0,10897	0,11382	0,10897	0,09850	0,10853	0,10853	0,10897	0,09850	0,10371	0,07791	0,06810	0,07791	0,07791	0,07275	0,06810	0,06349	0,05868	0,06349	0,06810	0,06349	0,06834	0,07791	0,07298	0,09850	0,08791	0,08288	0	0,01741	0,01300	0,05891	0,05891	0,05891	0,05412
Hap_51	0,22868	0,13901	0,12775	0,12279	0,09375	0,08354	0,09893	0,09850	0,08354	0,09375	0,08862	0,10945	0,07852	0,08862	0,08825	0,08354	0,09850	0,09375	0,09375	0,09893	0,09893	0,09893	0,09335	0,09375	0,09850	0,09375	0,09375	0,09335	0,09335	0,09375	0,08354	0,08862	0,08320	0,07325	0,08320	0,08320	0,07791	0,07325	0,05891	0,05412	0,05891	0,05868	0,05891	0,05412	0,06349	0,05868	0,08354	0,07325	0,06834	0,01741	0	0,00431	0,05435	0,06401	0,06401	0,05916
Hap_52	0,22239	0,14453	0,12243	0,11750	0,08862	0,08862	0,10416	0,10371	0,08862	0,09893	0,09375	0,11480	0,08354	0,09375	0,09335	0,08862	0,10371	0,09893	0,09893	0,10416	0,10416	0,10416	0,09850	0,09893	0,10371	0,09893	0,09893	0,09850	0,09850	0,09893	0,08862	0,09375	0,08825	0,07820	0,08825	0,08825	0,08288	0,07820	0,06374	0,05891	0,06374	0,06349	0,06374	0,05891	0,06834	0,06349	0,08862	0,07820	0,07325	0,01300	0,00431	0	0,05916	0,06892	0,06892	0,06401
Hap_53	0,23505	0,12814	0,14961	0,14453	0,08354	0,07354	0,07852	0,07820	0,06374	0,07354	0,06861	0,08862	0,06861	0,07852	0,07820	0,07354	0,07820	0,07354	0,07354	0,06861	0,06861	0,06861	0,06834	0,06374	0,07820	0,07354	0,06374	0,07325	0,07325	0,07354	0,06374	0,06861	0,08825	0,08825	0,07820	0,08825	0,08288	0,07820	0,06374	0,06861	0,08354	0,07325	0,07354	0,05891	0,06349	0,05868	0,06861	0,06349	0,05868	0,05891	0,05435	0,05916	0	0,02644	0,02644	0,02193
Hap_54	0,22868	0,14453	0,15523	0,15012	0,09375	0,08354	0,08862	0,08825	0,07354	0,08354	0,07852	0,07852	0,08862	0,09893	0,09850	0,09375	0,09850	0,09375	0,08354	0,08862	0,07852	0,08862	0,08825	0,08354	0,09850	0,09375	0,08354	0,09335	0,09335	0,09375	0,08354	0,08862	0,08825	0,08825	0,07820	0,08825	0,08288	0,07820	0,07354	0,06861	0,08354	0,07325	0,07354	0,06861	0,08320	0,07820	0,07852	0,07325	0,06834	0,05891	0,06401	0,06892	0,02644	0	0,00866	0,00431
Hap_55	0,22868	0,14453	0,15523	0,15012	0,09375	0,08354	0,08862	0,08825	0,07354	0,08354	0,07852	0,07852	0,08862	0,09893	0,09850	0,09375	0,09850	0,09375	0,08354	0,08862	0,07852	0,08862	0,08825	0,08354	0,09850	0,09375	0,08354	0,09335	0,09335	0,09375	0,08354	0,08862	0,08825	0,08825	0,07820	0,08825	0,08288	0,07820	0,07354	0,06861	0,08354	0,07325	0,07354	0,06861	0,08320	0,07820	0,07852	0,07325	0,06834	0,05891	0,06401	0,06892	0,02644	0,00866	0	0,00431
Hap_56	0,22239	0,13901	0,14961	0,14453	0,08862	0,07852	0,08354	0,08320	0,06861	0,07852	0,07354	0,07354	0,08354	0,09375	0,09335	0,08862	0,09335	0,08862	0,07852	0,08354	0,07354	0,08354	0,08320	0,07852	0,09335	0,08862	0,07852	0,08825	0,08825	0,08862	0,07852	0,08354	0,08320	0,08320	0,07325	0,08320	0,07791	0,07325	0,06861	0,06374	0,07852	0,06834	0,06861	0,06374	0,07820	0,07325	0,07354	0,06834	0,06349	0,05412	0,05916	0,06401	0,02193	0,00431	0,00431	0

REFERENCES

1. Justi SA, Russo CA, Mallet JR, Rocha MN, Nogueira NB, Galvão C. Molecular phylogeny of Triatomini (Hemiptera: Reduviidae: Triatominae). Parasit Vectors. 2014; 7: 149.
2. Pessoa GCDÁ, de Sousa TN, Sonoda IV, Diotaiuti L. Assessing the mitochondrial DNA diversity of the Chagas disease vector *Triatoma sordida* (Hemiptera: Reduviidae). Mem Inst Oswaldo Cruz. 2016; 111(5): 322-9.
3. Gardim S, Almeida CE, Takiya DM, Oliveira J, Araújo RF, Cicarelli RM, et al. Multiple mitochondrial genes of some sylvatic Brazilian *Triatoma*: non-monophyly of the *T. brasiliensis* subcomplex and the need for a generic revision in the Triatomini. Infect Genet Evol. 2014; 23: 74-9.
4. Madeira FF, Delgado LMG, Bittinelli IDF, Carvalho DB, Abreu MG, Azeredo-Oliveira MTV, et al. *Triatoma sordida* (Hemiptera, Triatominae) from La Paz, Bolivia: an incipient species or an intraspecific chromosomal polymorphism? Parasit Vectors. 2021; 14: 553.
5. Waleckx E, Salas R, Huaman N, Buitrago R, Bosseno MF, Aliaga C, et al. New insights on the Chagas disease main vector *Triatoma infestans* (Reduviidae, Triatominae) brought by the genetic analysis of Bolivian sylvatic populations. Infect Genet Evol. 2011; 11(5): 1045-57.
6. Lyman ED, Monteiro FA, Escalante AA, Cordon-Rosales C, Wesson DM, Dujardin J-P, et al. Mitochondrial DNA sequence variation among triatomine vectors of Chagas' disease. Am J Trop Med Hyg. 1999; 60: 377-86.
